# Supplementary material for: The E3 Ubiquitin Ligase ATL9 Affects Expression of Defense Related Genes, Cell Death and Callose Deposition in Response to Fungal Infection
Source: Pathogens. 2022 Jan 5;11(1):68. doi: 10.3390/pathogens11010068 (PMC8778023; doi:10.3390/pathogens11010068)
Supplement: Supplementary file 1 [file pathogens-11-00068-s001.zip › pathogens-1491921- Supplementary materials.pdf]

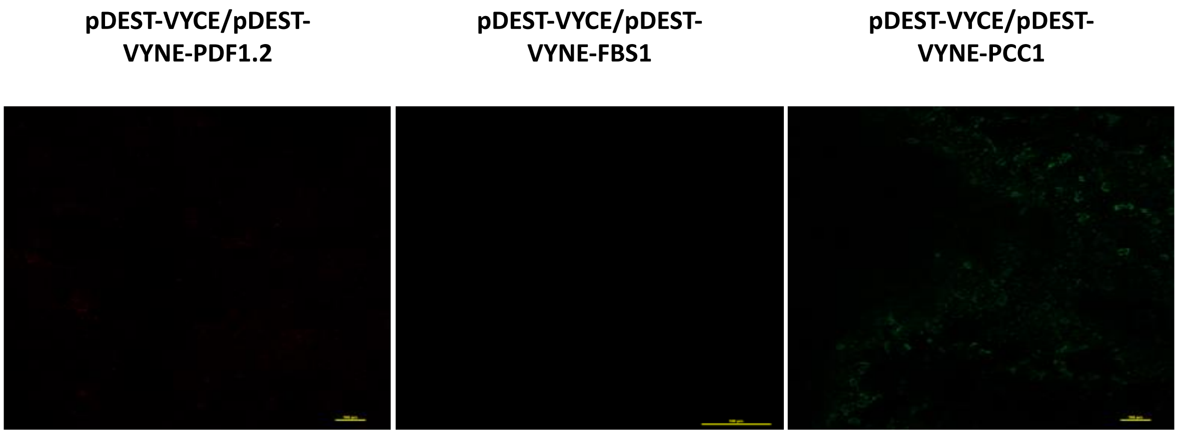

Figure S1. Negative control of bimolecular fluorescence complementation assay. pDEST-VYCE was transiently coexpressed with pDEST-VYNE-FBS1, pDEST-VYNE-PCC1, and pDEST-VYNE-PDF1.2 in tobbaco leaves. Fluorescence emission was observed under the confocal microscope. Results showed that yellow fluorescence is not caused by the vector used for cloning *ATL9*.

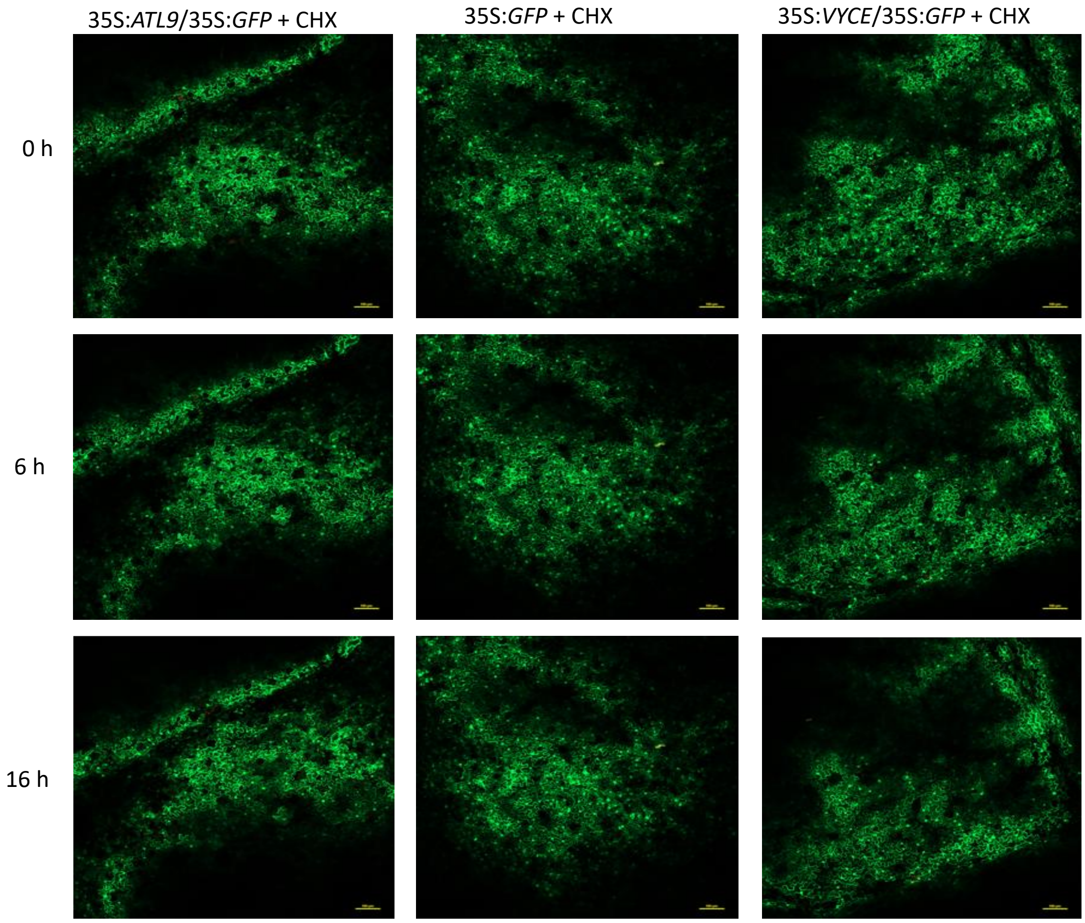

Figure S2. Controls used in in vivo ubiquitination Assay. To examine whether degradation could be caused by the vectors used in the experiment, 35S:ATL9 was transiently co-expressed with 35S:GFP in N. benthamiana. In addition, 35S:VYCE used to construct the overexpression ATL9 line was co-transformed with 35S:GFP in N. benthamiana. We also investigated whether 35S:GFP would degrade on its own

under experimental conditions. After 36 hours incubation, leaves were treated with either 100 μM CHX or 100 μM CHX plus 100 μM MG132 before observation and then fluorescence emission was monitored using confocal microscope.
